# A New Method for Precision Measurement of Wall-Thickness of Thin-Walled Spherical Shell Parts

**DOI:** 10.3390/mi12050467

**Published:** 2021-04-21

**Authors:** Jiang Guo, Yongbo Xu, Bo Pan, Juntao Zhang, Renke Kang, Wen Huang, Dongxing Du

**Affiliations:** 1 Key Laboratory for Precision and Non-Traditional Machining Technology of Ministry of Education, Dalian University of Technology, Dalian 116024, China; guojiang@dlut.edu.cn (J.G.); xuyongbo130@163.com (Y.X.); panbo723@mail.dlut.edu.cn (B.P.); 15207144627@163.com (J.Z.); kangrk@dlut.edu.cn (R.K.); 2 Institute of Mechanical Manufacturing Technology, China Academy of Engineering Physics, Mianyang 621999, China; huangw0673@yinhe596.cn

**Keywords:** spherical shell, thin-walled part, wall-thickness, benchmark coincidence, data processing

## Abstract

Thin-walled parts are widely used in shock wave and detonation physics experiments, which require high surface accuracy and equal thickness. In order to obtain the wall thickness of thin-walled spherical shell parts accurately, a new measurement method is proposed. The trajectories, including meridian and concentric trajectories, are employed to measure the thickness of thin-walled spherical shell parts. The measurement data of the inner and outer surfaces are unified in the same coordinate system, and the thickness is obtained based on a reconstruction model. The meridian and concentric circles’ trajectories are used for measuring a spherical shell with an outer diameter of Φ210.6 mm and an inner diameter of Φ206.4 mm. Without the data in the top area, the surface errors of the outer and inner surfaces are about 5 μm and 6 μm, respectively, and the wall-thickness error is about 8 μm with the meridian trajectory.

## 1. Introduction

Thin-walled parts are widely used in aerospace equipment, such as rocket tank panels, rocket nozzles and aircraft skins [1,2]. Spherical thin-walled parts have typical geometric shapes, and the mathematical description of their deformation and failure is easy to express. In terms of physical design, spherical thin-walled parts take advantage of “high energy efficiency and low energy consumption”, and the spherical thin-walled parts are often used in shock wave physics, and are used to develop the mechanism of various physical, chemical and mechanical phenomena [3]. For example, spherical thin-walled parts are mostly used in the design of explosive physics experiment parts, where structures gather energy easily. By studying the deformation and destruction of the parts before and after the experiment, the important parameters that characterize the implosion process of the weapon are obtained. In order to improve the accuracy and reliability of important scientific experimental data of shock wave physics and detonation physics, the surface shape accuracy and wall-thickness uniformity of thin-walled parts of the spherical shell put forward extremely high requirements [3,4]. The machining of thin-walled freeform components has many challenges in terms of the geometrical complexity, high-requirement accuracy, and especially low rigidity [5]. The thin-walled parts have low rigidity and are difficult to clamp, and therefore it is hard to measure the thickness. For the wall-thickness measurement of curved parts, the main problem is that the wall-thickness information of the part needs to be obtained in the normal direction of a certain point.

At present, researchers have explored the wall-thickness measurement. The methods of measuring wall-thickness are divided into two kinds: direct measurement method and indirect measurement method. The main principle of the direct measurement method is to directly measure the distance between the inner and outer walls in the normal direction of the corresponding point as the point’s wall-thickness. Cao et al. [6] proposed a dual-probe contact sensor measurement scheme. By constructing a theoretical curve, the articulation center is established to always move along the motion curve, so that the connection between the fixed contact and the sensor probe is always in the normal direction of the inner wall to obtain the wall-thickness value of the workpiece. 

Wei et al. [7] proposed a method to measure the wall-thickness of seamless steel pipes in the normal direction using resistance strain sensors. Zhang et al. [8] used a photoelectric sensor probe to obtain wall-thickness information by ensuring that the line of the measuring clamp between the contact point on the inner wall and the contact point on the outer wall is always in the normal direction of the measured point. Guo et al. [9] developed a special geometric parameter measuring instrument based on the photoelectric micro-displacement sensor, which can directly measure the normal geometric thickness of thin-walled parts with deep holes. Lyssakow et al. [10] used two laser sensors to successfully obtain the geometric defects and thickness defects of the cylindrical structure. Jin et al. [11] proposed a tubing wall-thickness measurement method based on a measurement sensor and designed an online tubing wall-thickness measurement system with a wall-thickness measurement accuracy of ±0.05 mm. The indirect measurement method is mainly based on the principle of eddy current measurement and ultrasonic measurement, and the wall-thickness information is calculated by the indirect quantity related to the wall-thickness. Generally, the use of eddy current to measure the wall-thickness of a part is to obtain the thickness information by extracting the peak value and peak time in the pulse eddy current response. Fan et al. [12] proposed the spectral pulse eddy current response of Hall sensors to obtain wall-thickness information. Mao et al. [13] proposed a method of using eddy currents to estimate the thickness of the pipeline, mainly by placing the excitation electromagnetic coil outside the pipeline to be tested, determining the relationship between the relative capacitance of the excitation coil and the pipe wall-thickness, and estimating the wall-thickness. Nishino et al. [14] proposed a method of measuring tube wall-thickness using ring wave resonance generated by a piezoelectric ring sensor, and verified through experiments that the wall-thickness error obtained by this method is less than 1.5%. Li et al. [15] proposed a pulse eddy current signal processing thickness measurement method for metal parts based on the Laplace wavelet characteristic frequency, and used 304 stainless steel specimens with a wall-thickness of 12 mm to 30 mm for experimental verification. The error of wall-thickness measurement results within the range of 0 mm to 120 mm in height is within 8%. Ultrasonic pulse echo technology is widely used in thickness measurement. Wu et al. [16] used the principle of ultrasonic reflection to measure the thickness of the pipeline by calculating the difference in the arrival time of the echo signal according to the arrival time of the echo signal. Jaime et al. [17] proposed a method where two orthogonally polarized shear waves were excited in the metal material by two mutually orthogonal coils, and the material thickness and crack defects were measured simultaneously in the pulse echo mode. Durongsak et al. [18] used the gamma rays obtain the wall-thickness information of the carbon steel pipe according to the energy change of the reflected ray. Levesque et al. [19] made the ultrasonic wave emit perpendicular to the surface. After the ultrasonic wave was reflected by the inner wall of the pipe, the wall-thickness of the pipe was obtained by detecting the wave caused by the reflected ultrasonic wave on the outer surface. Liu et al. [20] designed an ultrasonic scanning device to measure the thickness of large aerospace thin-walled parts, and used large aerospace aluminum alloy grid plates to verify the practicability of the device. In order to evaluate the measurement uncertainty of ultrasonic wall-thickness measurement, Morana et al. [21] used the Monte Carlo simulation method to establish a mathematical model for the estimation of ultrasonic measurement thickness uncertainty. In order to reduce the influence of the measurement environment on ultrasonic measurement, Adamowski et al. [22] proposed a temperature correction strategy for an ultrasonic measurement system, which can obtain the loss of micron wall-thickness due to internal corrosion of the pipeline, and monitor the corrosion of the pipeline for a long time. Rees et al. [23] obtained the thickness information of the glass furnace by measuring the microwave signal round-trip path, and improved the stability of the measurement by enhancing the echo signal at the receiving end.

The direct method is widely used to measure the wall-thickness, but the process is cumbersome and takes a long time. During the measurement, the connection between the inner and the outer wall contact point must always be in the normal direction of the measured point, which makes achieving a high-precision measurement difficult. The eddy current method to measure the wall-thickness needs a large amount of calculation and complex equipment. The measurement environment and the strength of the ultrasonic echo signal have a greater impact on the measurement result, and the cost of the ultrasonic device is relatively high. For spherical shell parts with micron-level precision, the above method is not suitable for the wall-thickness detection of spherical shell parts.

This paper proposes a method for measuring the wall-thickness of thin-walled spherical shell parts. The method is based on the three-coordinate measuring machine (CMM) with sub-micron measurement accuracy to obtain measurement data points. The measurement trajectory is planned for thin-walled spherical shell parts. By establishing a measurement reference coincidence model under the spatial coordinate system, the data on the inner and outer surfaces are unified to the same coordinate system. Based on the reconstruction model method of spherical shell parts, the wall-thickness information is obtained.

## 2. Methodology

### 2.1. The Method of Measuring Wall-Thickness

**Figure 1:**
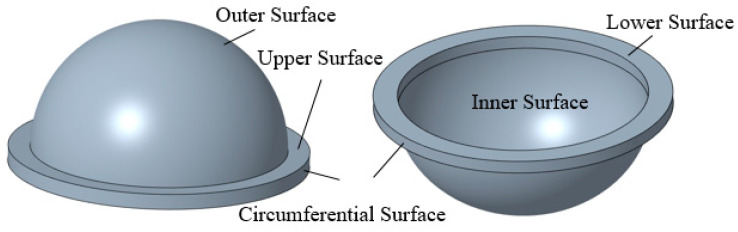
The spherical shell part.

**Figure 2:**
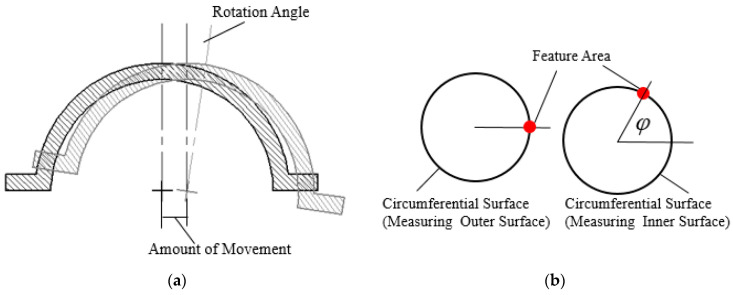
The schematic: (**a**) the amount of rotation and movement; (**b**) the rotation angle.

**Figure 3:**
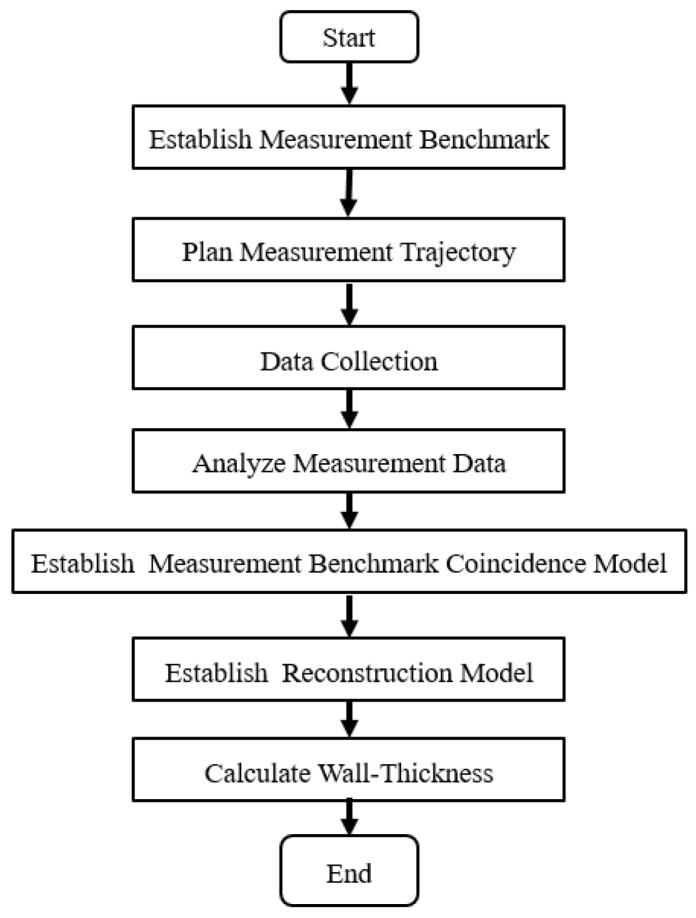
The measurement process.

The thin-walled spherical shell parts are shown in Figure 1. During the measurement, the workpiece experiences the overturn process, which will cause the inner and outer surfaces to have different axes. Furthermore, it will induce an error in the wall-thickness. Thus, to obtain the wall-thickness accurately, the rotation and movement along the x-axis, y-axis, and z-axis between the two positions shown in Figure 2 should be modified. Therefore, a new method for measuring the wall-thickness of thin-walled spherical shell parts is proposed. In the process of measuring the inner and outer surfaces of the spherical shell, the flange circumference is employed as the measurement benchmark. The model for the measurement benchmark coincidence in one spatial coordinate system is established. The reconstruction model of the spherical shell part is built based on the benchmark, and the wall-thickness is obtained. The measurement process is shown in Figure 3.

### 2.2. Measurement Trajectory

**Figure 4:**
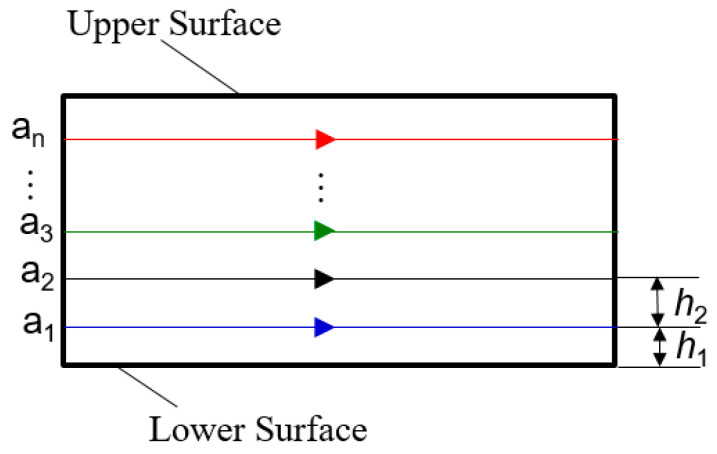
The circular measurement trajectory.

During the measurements, the spherical shell parts are placed on the measuring platform. The data on spherical shell parts and the points ($x_{\mathrm{wc}}$, $y_{\mathrm{wc}}$, $z_{\mathrm{wc}}$) on the flange circumference are obtained based on a precision coordinate measuring machine. As shown in Figure 4, the trajectory includes a circular trajectories, and the equation of the $n_{\mathrm{wc}}$-th circular trajectory can be expressed as:

(1) $$\{\begin{array}{c} x^{2}+y^{2}=R_{1}{}^{2}\\ z=h_{1}+(n_{\mathrm{wc}}-1)*h_{2} \end{array},0\leq z\leq h$$

where $R_{1}$ is the radius of the flange circumference, $h_{1}$ is the distance between the lower end of the flange and the first circular track, $h_{2}$ is the distance between each circular track, and h is the distance between the upper end of the flange and the lower end of the flange distance.

**Figure 5:**
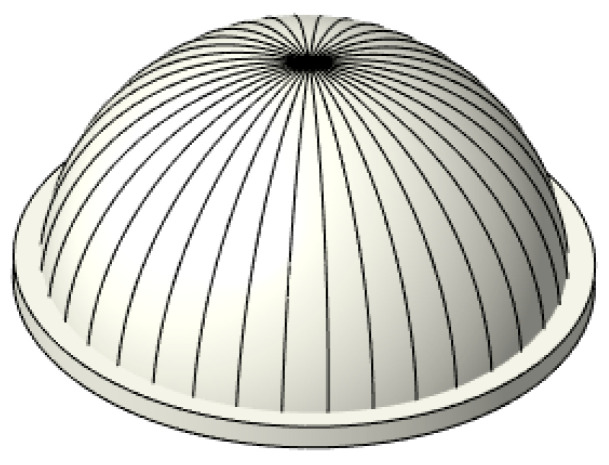
The meridian trajectory.

**Figure 6:**
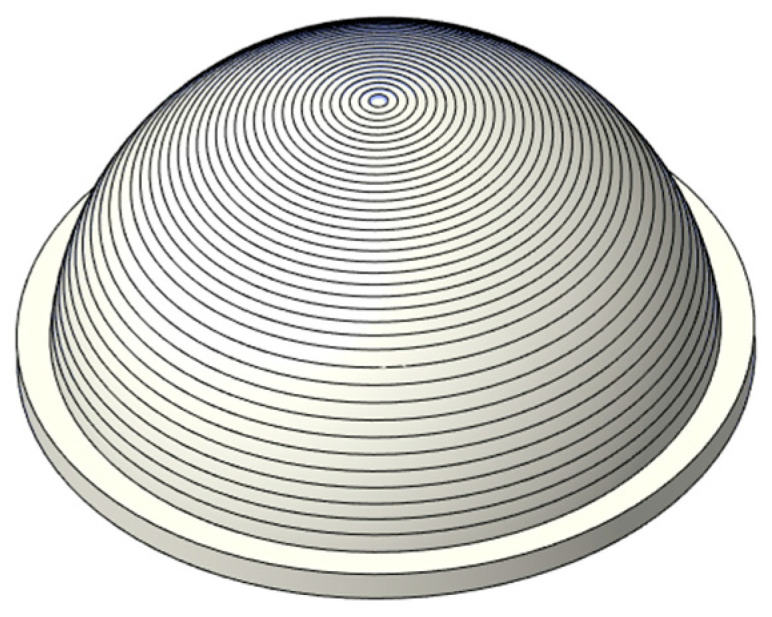
The concentric trajectory.

The meridian trajectory and concentric trajectory are shown in Figure 5 and Figure 6, respectively. The data points on the outer surface are ($x_{\mathrm{ww}1}$, $y_{\mathrm{ww}1}$, $z_{\mathrm{ww}1}$) and ($x_{\mathrm{wt}1}$, $y_{\mathrm{wt}1}$, $z_{\mathrm{wt}1}$); the equation of the $n_{\mathrm{ww}1}$ (≤b)-th meridian measurement track is:

(2) $$\{\begin{array}{c} x^{2}+y^{2}+z^{2}=R_{2}{}^{2}\\ y=x*\tan\left[\frac{2\pi \left(n_{\mathrm{ww}1}-1\right)}{b}\right] \end{array},0\leq x^{2}+y^{2}\leq R_{3}{}^{2}$$

The $n_{\mathrm{wt}1}$ (≤c)-th concentric circle measurement trajectory equation is:

(3) $$\{\begin{array}{c} x^{2}+y^{2}+z^{2}=R_{2}{}^{2}\\ z=h_{n_{\mathrm{wt}1}} \end{array},0\leq x^{2}+y^{2}\leq R_{3}{}^{2}$$

where $h_{n_{\mathrm{wt}1}}$ is the distance of the $n_{\mathrm{wt}1}$-th concentric circle measuring track from the lower end of the flange, $R_{2}$ is the radius of the outer surface of the spherical shell, and $R_{3}$ is the radius of the intersection of the outer surface of the spherical shell and the upper end of the flange.

Secondly, the workpiece is turned over and placed on the measuring platform with the inner surface facing upwards. Then, the circumference of the flange is measured, of which tracks are circular trajectories, and data points ($x_{\mathrm{nc}}$, $y_{\mathrm{nc}}$, $z_{\mathrm{nc}}$)on the flange circumference are obtained.

The trajectories are d meridian trajectory and e concentric trajectories, respectively. The data points are ($x_{\mathrm{nw}1}$, $y_{\mathrm{nw}1}$, $z_{\mathrm{nw}1}$) and ($x_{\mathrm{nt}1}$, $y_{\mathrm{nt}1}$, $z_{\mathrm{nt}1}$); the equation of the $n_{\mathrm{nw}1}$ (≤d)-th meridian measurement track in the workpiece coordinate system is:

(4) $$\{\begin{array}{c} x^{2}+y^{2}+z^{2}=R_{4}{}^{2}\\ y=x*\tan\left[\frac{2\pi \left(n_{\mathrm{nw}1}-1\right)}{d}\right] \end{array},0\leq x^{2}+y^{2}\leq R_{4}{}^{2}$$

Among them, the equation of the $n_{\mathrm{nt}1}$ (≤e)-th concentric circle measuring track is:

(5) $$\{\begin{array}{c} x^{2}+y^{2}+z^{2}=R_{4}{}^{2}\\ z=h_{n_{\mathrm{nt}1}} \end{array},0\leq x^{2}+y^{2}\leq R_{4}{}^{2}$$

where $h_{n_{\mathrm{nt}1}}$ is the distance of the $n_{\mathrm{nw}1}$-th concentric circle measurement track from the lower end of the flange, and $R_{4}$ is the radius of the inner surface of the spherical shell.

Before obtaining the surface shape distribution, the measurement data of the inner and outer surfaces of the spherical shell need to be preprocessed. The preprocessing includes removing the singular items in the data and smoothing. The data points obtained after preprocessing are ($x_{\mathrm{ww}2}$, $y_{\mathrm{ww}2}$, $z_{\mathrm{ww}2}$), ($x_{\mathrm{nw}2}$,$y_{\mathrm{nw}2}$,$z_{\mathrm{nw}2}$), ($x_{\mathrm{wt}2}$, $y_{\mathrm{wt}2}$, $z_{\mathrm{wt}2}$) and ($x_{\mathrm{nt}2}$, $y_{\mathrm{nt}2}$, $z_{\mathrm{nt}2}$). According to the data points after preprocessing, the sphere center of the outer surface of the meridian spherical shell is recorded as ($x_{\mathrm{ww}}$, $y_{\mathrm{ww}}$, $z_{\mathrm{ww}}$), the sphere radius is $R_{\mathrm{ww}}$, the center of the sphere on the inner surface of the meridian spherical shell is recorded as ($x_{\mathrm{nw}}$, $y_{\mathrm{nw}}$, $z_{\mathrm{nw}}$), and the sphere radius is $R_{\mathrm{nw}}$. The center of the sphere on the outer surface of the concentric spherical shell is recorded as ($x_{\mathrm{wt}}$, $y_{\mathrm{wt}}$, $z_{\mathrm{wt}}$), the radius of the sphere is $R_{\mathrm{wt}}$, the center of the sphere on the inner surface of the concentric spherical shell is recorded as ($x_{\mathrm{nt}}$, $y_{\mathrm{nt}}$, $z_{\mathrm{nt}}$) and the radius of the sphere is $R_{\mathrm{nt}}$. The error of the i-th data point position is on the surface. To obtain the surface error, the surface error formula is as follows:

(6) $$\left\{ \begin{array}{l} d_{\mathrm{ww}}\left(i\right)\;=\sqrt{{\left(x_{\mathrm{ww}2}-x_{\mathrm{ww}}\right)}^{2}+{\left(y_{\mathrm{ww}2}-y_{\mathrm{ww}}\right)}^{2}+{\left(z_{\mathrm{ww}2}-z_{\mathrm{ww}}\right)}^{2}}-R_{\mathrm{ww}}\\ d_{\mathrm{nw}}\left(i\right)\;=\sqrt{{\left(x_{\mathrm{nw}2}-x_{\mathrm{nw}}\right)}^{2}+{\left(y_{\mathrm{nw}2}-y_{\mathrm{nw}}\right)}^{2}+{\left(z_{\mathrm{nw}2}-z_{\mathrm{nw}}\right)}^{2}}\;-R_{\mathrm{nw}}\\ d_{\mathrm{wt}}\left(i\right)\;\;=\sqrt{{\left(x_{\mathrm{wt}2}-x_{\mathrm{wt}}\right)}^{2}+{\left(y_{\mathrm{wt}2}-y_{\mathrm{wt}}\right)}^{2}+{\left(z_{\mathrm{wt}2}-z_{\mathrm{wt}}\right)}^{2}}-R_{\mathrm{wt}}\\ d_{\mathrm{nt}}\left(i\right)\;\;=\sqrt{{\left(x_{\mathrm{nt}2}-x_{\mathrm{nt}}\right)}^{2}+{\left(y_{\mathrm{nt}2}-y_{\mathrm{nt}}\right)}^{2}+{\left(z_{\mathrm{nt}2}-z_{\mathrm{nt}}\right)}^{2}}-R_{\mathrm{nt}\;\;\;\;\;\;\;\;\;} \end{array}\right.$$

### 2.3. Measuring Benchmark Coincidence Model

In the process of obtaining data points on the inner and outer surfaces of spherical shell parts, the data points on the inner and outer surfaces are not in the same coordinate system due to the flip of the spherical shell. In order to calculate the wall-thickness information of spherical shell parts, it is necessary to make the data points in the same coordinate system.

When measuring the inner and outer surfaces, there may be rotations along the x-axis, y-axis and *z*-axis between the two spherical shell positions, which are α, β and θ respectively. Moreover, there is a rotation angle φ around its own axis. The movement along the x-axis and y-axis between the two spherical shell positions cannot be ignored. Since the reference in the z direction does not move during the turnover process, the movement between the x-axis and the y-axis can be acquired, which are and, respectively. In order to obtain these six parameters, it is necessary to obtain the rotation corresponding to the flange circumference data points ($x_{\mathrm{wc}}$, $y_{\mathrm{wc}}$, $z_{\mathrm{wc}}$) when measuring the outer surface, and the flange circumference data points ($x_{\mathrm{nc}}$, $y_{\mathrm{nc}}$, $z_{\mathrm{nc}}$) when measuring the inner surface. The new data points ($x_{\mathrm{wc}1}$, $y_{\mathrm{wc}1}$, $z_{\mathrm{wc}1}$) and ($x_{\mathrm{nc}1}$, $y_{\mathrm{nc}1}$, $z_{\mathrm{nc}1}$) are obtained by eliminating singularities in data points ($x_{\mathrm{wc}}$, $y_{\mathrm{wc}}$, $z_{\mathrm{wc}}$) and ($x_{\mathrm{nc}}$, $y_{\mathrm{nc}}$, $z_{\mathrm{nc}}$). The movement amounts m, n are obtained by processing the data. The points ($x_{\mathrm{wc}1}$, $y_{\mathrm{wc}1}$, $z_{\mathrm{wc}1}$) contain a circular trajectory and the points of the i-th circular trajectory are ($x_{\mathrm{wc}1i}$,$y_{\mathrm{wc}1i}$, $z_{\mathrm{wc}1i}$). The circle center ($x_{\mathrm{wci}}$, $y_{\mathrm{wci}}$, $z_{\mathrm{wci}}$) of the i-th circle track is obtained by fitting. The same method is used to obtain the circle center ($x_{\mathrm{nc}1}$, $y_{\mathrm{nc}1}$, $z_{\mathrm{nc}1}$). The horizontal and vertical coordinates of the flange axis for the first and second times are $m_{1}$, $n_{1}$, $m_{2}$ and $n_{2}$, respectively, which can be expressed as:

(7) $$\{\begin{array}{c} m_{1}=\frac{1}{a}*\sum_{i=1}^{a}x_{\mathrm{wc}}\left(i\right)\\ n_{1}=\frac{1}{a}*\sum_{i=1}^{a}y_{\mathrm{wc}}\left(i\right)\\ m_{2}=\frac{1}{a}*\sum_{i=1}^{a}x_{\mathrm{nc}}\left(i\right)\\ n_{2}=\frac{1}{a}*\sum_{i=1}^{a}y_{\mathrm{nc}}\left(i\right) \end{array}$$

where $h_{n_{\mathrm{nt}1}}$ is the distance of the $n_{\mathrm{nw}1}$-th concentric circle measurement track from the lower end of the flange, and $R_{4}$ is the radius of the inner surface of the spherical shell.

The amount of movement between x-axis and y-axis can be expressed as:

(8) $$\{\begin{array}{c} m=m_{1}-m_{2}\\ n=n_{1}-n_{2} \end{array}$$

The direct method is used to obtain the rotation angles α, β and θ. The direct method refers to matching the pre-processed flange data points ($x_{\mathrm{wc}1i}$, $y_{\mathrm{wc}1i}$, $z_{\mathrm{wc}1i}$) and ($x_{\mathrm{nc}1i}$, $y_{\mathrm{nc}1i}$, $z_{\mathrm{nc}1i}$) without fitting. The ranges of α, β are $\left[\alpha_{1},\alpha_{2}\right]$, $\left[\beta_{1},\beta_{2}\right]$, respectively. Take a points in the $\left[\alpha_{1},\alpha_{2}\right]$ range, and the i point is $\alpha_{i}$; in the $\left[\beta_{1},\beta_{2}\right]$ range, take b points, and the j point is $\beta_{j}$. Take $\alpha_{i}$ and $\beta_{j}$ as a set of data and record them as ($\alpha_{i}$, $\beta_{j}$) and a∗b can be taken in total group data, where:

(9) $$\{\begin{array}{c} \alpha_{i}=\alpha_{1}+\frac{1}{\alpha }*\left(i-1\right)*\left(\alpha_{2}-\alpha_{1}\right)\\ \beta_{i}=\beta_{1}+\frac{1}{\beta }*\left(j-1\right)*\left(\beta_{2}-\beta_{1}\right) \end{array}$$

The flange data points ($x_{\mathrm{nc}3i}$, $y_{\mathrm{nc}3i}$, $z_{\mathrm{nc}3i}$) are obtained by coordinate conversion. The formula is as follows:

(10) $$\left[\begin{array}{c} x_{\mathrm{nc}2i}\\ y_{\mathrm{nc}2i}\\ z_{\mathrm{nc}2i} \end{array}\right]=\left(\begin{array}{ccc} 1 & 0 & 0\\ 0 & \cos\;\alpha_{i} & \sin\;\alpha_{i}\\ 0 & \sin\;\alpha_{i} & \cos\;\alpha_{i} \end{array}\right)\left[\begin{array}{c} x_{\mathrm{nc}1i}\\ y_{\mathrm{nc}1i}\\ z_{\mathrm{nc}1i} \end{array}\right]$$

(11) $$\left[\begin{array}{c} x_{\mathrm{nc}3i}\\ y_{\mathrm{nc}3i}\\ z_{\mathrm{nc}3i} \end{array}\right]=\left(\begin{array}{ccc} \cos\;\beta_{j} & 0 & -\sin\;\beta_{j}\\ 0 & 1 & 0\\ \sin\;\beta_{j} & 0 & \cos\;\beta_{j} \end{array}\right)\left[\begin{array}{c} x_{\mathrm{nc}2i}\\ y_{\mathrm{nc}2i}\\ z_{\mathrm{nc}2i} \end{array}\right]$$

After the rotation of $\alpha_{i}$ and $\beta_{j}$, the coincidence error between the corresponding points is recorded as $t_{ij}$. The formula is as follows:

(12) $$t_{ij}=\sum_{i=1}^{u}\sqrt{{\left(x_{\mathrm{wc}1i}-x_{\mathrm{nc}3i}\right)}^{2}+{\left(y_{\mathrm{wc}1i}-y_{\mathrm{nc}3i}\right)}^{2}+{\left(z_{\mathrm{wc}1i}-z_{\mathrm{nc}3i}\right)}^{2}}$$

The minimum value $t_{0}$ is obtained by comparing $t_{11},t_{12}\ldots t_{\mathrm{ab}}$. Under this condition, the rotation angle $\alpha_{i}$ around the x axis is recorded as α, the rotation angle $\beta_{j}$ around the y axis is recorded as $\beta_{0}$, and ($x_{\mathrm{nc}4i}$, $y_{\mathrm{nc}4i}$, $z_{\mathrm{nc}4i}$) is recorded as ($x_{\mathrm{nc}5i}$, $y_{\mathrm{nc}5i}$, $z_{\mathrm{nc}5i}$).

Data points ($x_{\mathrm{wc}1i}$, $y_{\mathrm{wc}1i}$, $z_{\mathrm{wc}1i}$) and ($x_{\mathrm{nc}5i}$, $y_{\mathrm{nc}5i}$, $z_{\mathrm{nc}5i}$) contain u data points. The distance $d_{w}$ from each data point in ($x_{\mathrm{wc}1i}$,$y_{\mathrm{wc}1i}$,$z_{\mathrm{wc}1i}$) to the center ($x_{\mathrm{wci}}$, $y_{\mathrm{wci}}$, $z_{\mathrm{wci}}$) in the data is recorded as $d_{w1}$, $d_{w2}$ ……$d_{\mathrm{wu}}$. The distance $d_{n}$ from each data point in ($x_{\mathrm{nc}5i}$, $y_{\mathrm{nc}5i}$, $z_{\mathrm{nc}5i}$) to the center ($x_{\mathrm{nci}}$, $y_{\mathrm{nci}}$, $z_{\mathrm{nci}}$) in the data is recorded in turn as $d_{n1},d_{n2}\ldots d_{\mathrm{nu}}$. To calculate the arithmetic sum of squares of the errors in different correspondences, the formula is as follows:

(13) $$\left\{ \begin{array}{l} \Delta_{1}\;\;\;={\left(d_{w1}-d_{n1}\right)}^{2}+{\left(d_{w1}-d_{n1}\right)}^{2}+\cdots +{\left(d_{\mathrm{wu}}-d_{\mathrm{nu}}\right)}^{2}\\ \Delta_{2}\;\;\;={\left(d_{w1}-d_{n2}\right)}^{2}+{\left(d_{w1}-d_{n3}\right)}^{2}+\cdots +{\left(d_{w\left(u-1\right)}-d_{\mathrm{nu}}\right)}^{2}+{\left(d_{\mathrm{wu}}-d_{n1}\right)}^{2}\\ \;\;\;\;\;\;\;\;\;\;\;\;\;\;\;\;\;\;\;\;\;\;\;\ldots \\ \Delta_{u-1}={\left(d_{w1}-d_{\mathrm{nu}}\right)}^{2}+{\left(d_{w2}-d_{n1}\right)}^{2}+\cdots +{\left(d_{w\left(u-1\right)}-d_{n\left(u-2\right)}\right)}^{2}+{\left(d_{\mathrm{wu}}-d_{n\left(u-1\right)}\right)}^{2} \end{array}\right.$$

The minimum value $\Delta_{i}$ is obtained by comparing $\Delta_{1},\Delta_{2}\ldots \Delta_{u-1}$, and the rotation angle φ along the z-axis is obtained as follows:

(14) $$\varphi =\frac{i}{u}*2\pi$$

### 2.4. Reconstruction Model and Method of Obtaining Wall-Thickness

In order to obtain the wall-thickness of spherical shell parts, it is necessary to move and rotate the measurement data so that the inner and outer surfaces are unified under the same coordinate system. The subsequent processing method is the same, whether the meridian or concentric trajectory are used in the measurement. Thus, the meridian trajectory was taken as an example for wall-thickness processing. First, the external surface data points ($x_{\mathrm{ww}2}$, $y_{\mathrm{ww}2}$, $z_{\mathrm{ww}2}$) were processed. The formula is as follows:

(15) $$\left[\begin{array}{c} x_{\mathrm{ww}3}\\ y_{\mathrm{ww}3}\\ z_{\mathrm{ww}3} \end{array}\right]=\left[\begin{array}{c} x_{\mathrm{ww}2}\\ y_{\mathrm{ww}2}\\ z_{\mathrm{ww}2} \end{array}\right]-\left[\begin{array}{c} m_{1}\\ n_{1}\\ 0 \end{array}\right]$$

After preprocessing, the inner surface data points ($x_{\mathrm{nw}2}$, $y_{\mathrm{nw}2}$, $z_{\mathrm{nw}2}$) and ($x_{\mathrm{nt}2}$, $y_{\mathrm{nt}2}$, $z_{\mathrm{nt}2}$) are translated and rotated. The formula is as follows:

(16) $$\left[\begin{array}{c} x_{\mathrm{nw}3}\\ y_{\mathrm{nw}3}\\ z_{\mathrm{nw}3} \end{array}\right]=\left[\begin{array}{c} x_{\mathrm{nw}2}\\ y_{\mathrm{nw}2}\\ z_{\mathrm{nw}2} \end{array}\right]-\left[\begin{array}{c} m\\ n\\ 0 \end{array}\right]$$

(17) $$\left[\begin{array}{c} x_{\mathrm{nw}4}\\ y_{\mathrm{nw}4}\\ z_{\mathrm{nw}4} \end{array}\right]=\left(\begin{array}{ccc} 1 & 0 & 0\\ 0 & \cos\;\alpha_{0} & \sin\;\alpha_{0}\\ 0 & -\sin\;\alpha_{0} & \cos\;\alpha_{0} \end{array}\right)\left[\begin{array}{c} x_{\mathrm{nw}3}\\ y_{\mathrm{nw}3}\\ z_{\mathrm{nw}3} \end{array}\right]$$

(18) $$\left[\begin{array}{c} x_{\mathrm{nw}5}\\ y_{\mathrm{nw}5}\\ z_{\mathrm{nw}5} \end{array}\right]=\left(\begin{array}{ccc} \cos\;\beta_{0} & 0 & -\sin\;\beta_{0}\\ 0 & 1 & 0\\ \sin\;\beta_{0} & 0 & \cos\;\beta_{0} \end{array}\right)\left[\begin{array}{c} x_{\mathrm{nw}4}\\ y_{\mathrm{nw}4}\\ z_{\mathrm{nw}4} \end{array}\right]$$

(19) $$\left[\begin{array}{c} x_{\mathrm{nw}6}\\ y_{\mathrm{nw}6}\\ z_{\mathrm{nw}6} \end{array}\right]=\left(\begin{array}{ccc} \cos\;\theta_{0} & \sin\;\theta_{0} & 0\\ -\sin\;\theta_{0} & \cos\;\theta_{0} & 0\\ 0 & 0 & 1 \end{array}\right)\left[\begin{array}{c} x_{\mathrm{nw}5}\\ y_{\mathrm{nw}5}\\ z_{\mathrm{nw}5} \end{array}\right]$$

The outer surface points ($\theta_{w}$, $\phi_{w}$, $r_{w}$) and the inner surface points ($\theta_{n}$, $\phi_{n}$, $r_{n}$) are obtained by coordinate conversion. The conversion formula is as follows:

(20) $$\{\begin{array}{c} r=\sqrt{x^{2}+y^{2}+z^{2}}\\ \theta =\cos^{-1}\left(\frac{z}{\sqrt{x^{2}+y^{2}+z^{2}}}\right)\\ \phi =\tan^{-1}\frac{y}{x} \end{array}$$

In the interpolation, θ is −180°~180°, the interpolation interval is Δθ; ϕ is 0°~90°, and the interpolation interval is Δϕ; The outer surface interpolation point ($\theta_{e}$, $\phi_{e}$, $r_{\mathrm{we}}$) and the inner surface interpolation point ($\theta_{e}$, $\phi_{e}$, $r_{\mathrm{ne}}$) are obtained according to data points ($\theta_{w}$, $\phi_{w}$, $r_{w}$) and ($\theta_{n}$, $\phi_{n}$, $r_{n}$), and the wall-thickness difference formula as:

(21) $$h_{e}=r_{\mathrm{we}}-r_{\mathrm{ne}}$$

## 3. Experimental Setup

**Figure 7:**
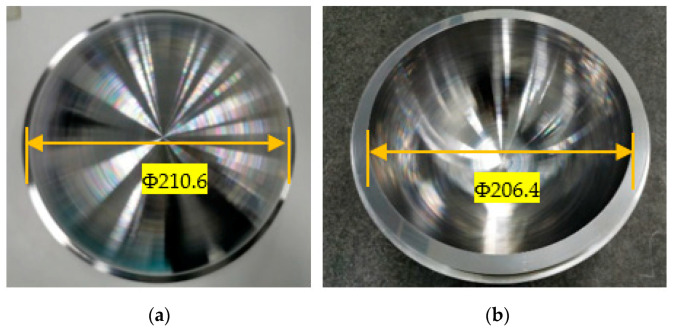
The processed spherical shell: (**a**) the outer surface; (**b**) the inner surface.

A spherical shell with an outer diameter of Φ210.6 mm and an inner diameter of Φ206.4 mm is used for the measurement test. As shown in Figure 7, the processing machine is an ultra-precision single-point diamond machine made by Precitech in the United States. The machine parameters are shown in Table 1. The cemented carbide tool (KC5010) is used for processing and the constant speed is used for machining. The specific parameters are shown in Table 2. The spherical shell is measured with CMM by Zeiss, Germany. The maximum allowable error of the size measurement is ±0.5 + *L*/500 μm, and *L* is the length to be measured. For the most part, the measurement accuracy is about 1 μm. A ruby probe with a diameter of 5 mm is used in the processing.

**Figure 8:**
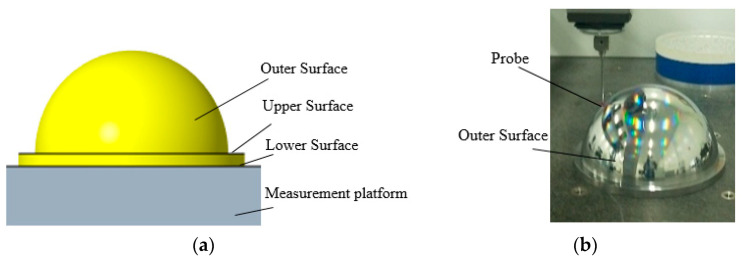
The outer surface measurement: (**a**) the measurement model; (**b**) measuring the real object.

The circular trajectory on the circumferential surface of the spherical shell flange is measured first, and the spherical shell is placed on the three-coordinate platform with the outer surface facing upwards, as shown in Figure 8. The data point ($x_{\mathrm{wc}}$, $y_{\mathrm{wc}}$, $z_{\mathrm{wc}}$) is measured according to the trajectory of the flange circumferential surface, which takes four circular trajectories. In the circular trajectory Equation (1), $R_{1}$ is 115 mm, $h_{1}$ is 4 mm, $h_{2}$ is 2 mm, and h is 12 mm.

**Figure 9:**
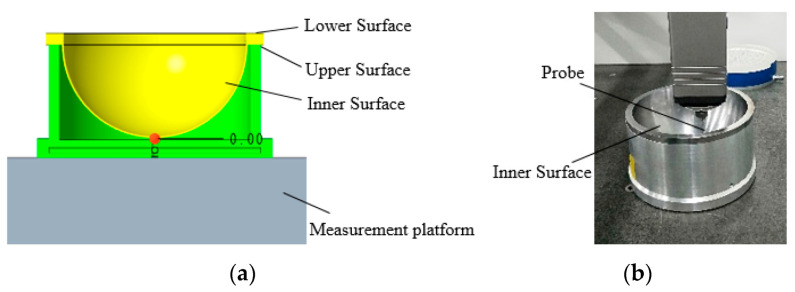
The inner surface measurement: (**a**) the measurement model; (**b**) measuring the real object.

The outer surface is measured according to the meridian and concentric circles. The meridian measurement of the outer surface takes 18 meridian trajectories. In Equation (2), $R_{2}$ is 105.3 mm, $R_{4}$ is 103.2 mm, and b is 18. The obtained data points are ($x_{\mathrm{ww}1}$, $y_{\mathrm{ww}1}$, $z_{\mathrm{ww}1}$). Concentric circle traces of 28 concentric circles are taken to obtain data point ($x_{\mathrm{wt}1}$, $y_{\mathrm{wt}1}$, $z_{\mathrm{wt}1}$). Then the spherical shell is placed on the three-coordinate platform with the inner surface facing upwards, as shown in Figure 9. The data points ($x_{\mathrm{nc}}$, $y_{\mathrm{nc}}$, $z_{\mathrm{nc}}$) on the circumferential surface of the flange are obtained. Finally, the inner surface is measured according to the meridian and concentric circles. The meridian measurement of the inner surface takes 18 meridian trajectories to obtain the measurement data points ($x_{\mathrm{nw}1}$, $y_{\mathrm{nw}1}$, $z_{\mathrm{nw}1}$). The concentric circular measurement of the inner surface takes 29 measurement trajectories to obtain the measurement data points ($x_{\mathrm{nt}1}$, $y_{\mathrm{nt}1}$, $z_{\mathrm{nt}1}$).

## 4. Results and Discussion

### 4.1. Spherical Shell Surface Shape

**Figure 10:**
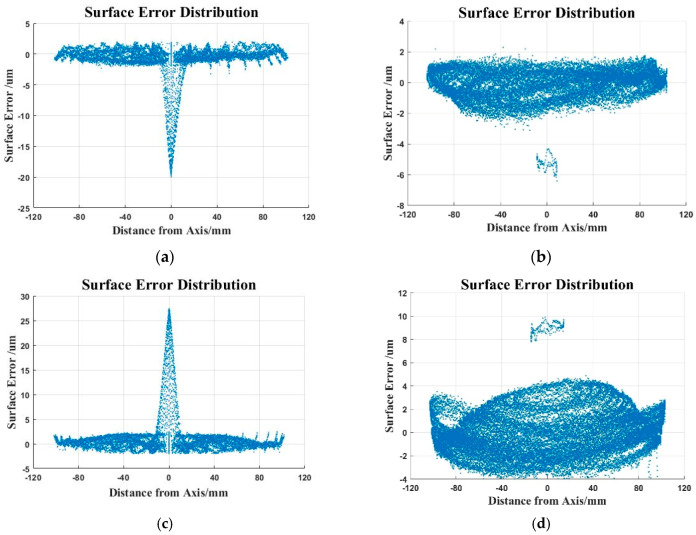
The surface error distribution: (**a**) Surface error distribution of the outer surface (the meridian trajectory); (**b**) surface error distribution of the outer surface (the concentric trajectory); (**c**) surface error distribution of the inner surface (the meridian trajectory); (**d**) surface error distribution of the inner surface (the concentric trajectory).

The surface errors of the outer and inner surface of the spherical shell near the top are about 22 μm and 27 μm respectively by the meridian trajectory, while the outer and inner surface errors near the top are about 8 μm and 14 μm by concentric circles. Due to the large error near the top, the surface error was evaluated without the points near the top. The results revealed that the errors of the inner and outer surfaces are 5 μm and 6 μm respectively by the meridian trajectory. Similar to the meridian trajectory, the inner and outer surfaces are 5 μm and 8 μm using concentric circles, as shown in Figure 10.

### 4.2. Wall-Thickness of Spherical Shell

When evaluating the wall-thickness, it is necessary to make the measurement benchmark coincide. According to Formula (7) and Formula (8), $m_{1}$, $m_{2}$, $n_{1}$, $n_{2}$, m and n are obtained, as shown in Table 3.

**Figure 11:**
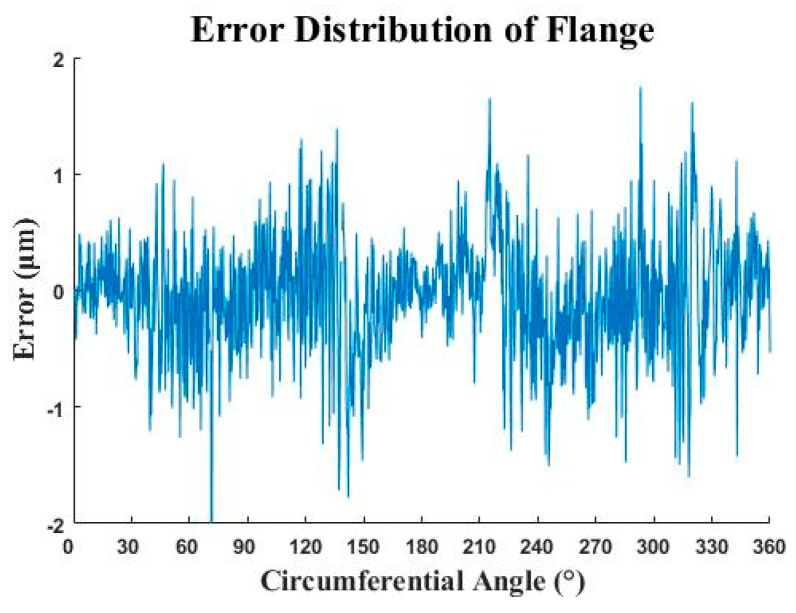
The error distribution of flange.

The direct method was used to obtain the rotation angles α, β and θ along the x-axis, y-axis and z-axis, which is $\alpha_{0}=0.07{}^{\circ}$, $\beta_{0}=0.26{}^{\circ}$, φ=17.67°. After moving the second flange data, the distance between the corresponding points was calculated as the error based on the rotation. The error of the flange circumference is less than 2 µm obviously, as shown in Figure 11.

**Figure 12:**
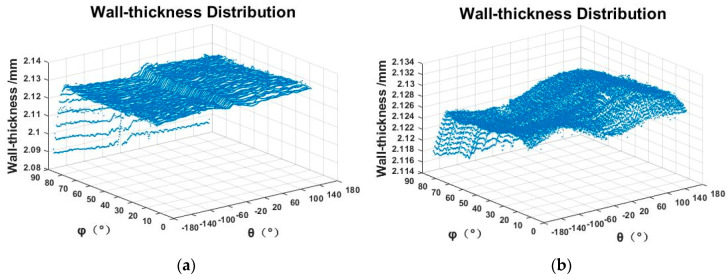
The wall-thickness distribution (polar coordinates): (**a**) the wall-thickness distribution (the meridian trajectory); (**b**) the wall-thickness distribution (the concentric trajectory).

The inner and outer surface data were rotated and translated according to Equations (15)–(19), and then interpolated in the inner and outer surfaces. The interpolation interval Δθ is 0.01° and Δϕ is 0.01°. According to Formula (21), the wall-thickness $h_{e}$ of each point is obtained. ($\theta_{e}$, $\phi_{e}$, $h_{e}$) are used as the data points to draw the spherical shell wall-thickness error distribution, as shown in Figure 12. The meridian measurement track is used to measure the wall-thickness error of about 45 μm. The concentric circle measurement track used to measure the wall-thickness error is about 16 μm.

**Figure 13:**
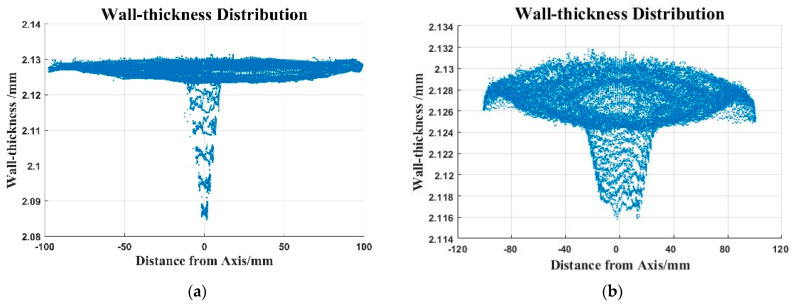
The wall-thickness distribution (Cartesian coordinates): (**a**) the wall-thickness distribution (the meridian trajectory); (**b**) the wall-thickness distribution (the concentric trajectory).

In order to describe the wall-thickness clearly, the interpolation data point ($\theta_{w}$, $\phi_{w}$, $r_{w}$) on the outer surface of the spherical shell is converted into rectangular coordinates ($x_{w}$, $y_{w}$, $z_{w}$), and the ($x_{w}$, $y_{w}$, $h_{e}$) is used as the data point to draw the spherical shell wall-thickness distribution, as shown in Figure 13.

### 4.3. Discussion

The surface error of the top by the meridian trajectory is larger than that of the concentric circles. This is because the measurement of the concentric circles fails to collect the top data information. In actual processing, due to the poor cutting conditions at the top area, the presence of entangled chips and a long distance from the positioning surface (flange) will cause a large wall-thickness error, and the uniformity of the wall-thickness at the top is more difficult to achieve. The meridian trajectory can reflect the wall-thickness information accurately. Hence, the meridian trajectory is more suitable for measuring the spherical shell part thickness than the concentric circle trajectory measurement. The wall-thickness difference is large near the top, and the error increased simultaneously. During the actual processing of the inner and outer surfaces, the workpiece is always machined from the flange position to the top, and the rigidity is weak in the top area. Thus, the surface error of the spherical shell is large and the wall-thickness uniformity is poor. There are several reasons for causing the problem, including built-up edge in the processing process, the deviation of tool installation center and rotation center of the workpiece, tool setting errors in the x and z directions, the harsh processing conditions, etc.

## 5. Conclusions

This paper proposes a method for measuring the wall-thickness of thin-walled spherical shell parts. Experiments are conducted on a spherical shell part with an outer diameter of Φ210.6 mm and an inner diameter of Φ206.4 mm. The meridian track and the concentric circle track are used to measure the spherical shell.

1. Thin-walled spherical shell parts thickness is obtained by measuring the surface of inner and outer surface respectively.
2. The surface error of the outer and inner surfaces of the spherical shell are about 5 μm and 6 μm, and the wall-thickness error is about 8 μm.
3. The meridian trajectory is verified as a better method to obtain the wall-thickness of spherical shell parts.

The measurement method is suitable for the wall-thickness of thin-walled spherical shell parts, and it has certain significance for subsequent scholars to study the thickness of thin-walled parts.

## Figures and Tables

**Table 1:** The machine parameters.

Parameter Type	Parameter Value
X Stroke (mm)	350
Z Stroke (mm)	300
Position Feedback Accuracy (nm)	0.032
X Horizontal Straightness (μm/25 mm)	0.05
Z Horizontal Straightness (μm/25 mm)	0.05
Spindle Load (kg)	85
Spindle Radial Runout (nm)	≤15
Spindle Axial Runout (nm)	≤15
Maximum Spindle Speed (rpm)	7000

**Table 2:** The processing related parameters.

Parameter Type	Parameter Value
Tool Radius (mm)	0.2
Spindle Speed (rpm)	200
F (mm/min)	20
ap (μm)	10
Adsorption Pressure (kPa)	50

**Table 3:** The related parameters.

Parameter	$m_{1}$	$m_{2}$	$n_{1}$	$n_{2}$	m	n
(μm)	−2.9486	1.7417	−0.4585	−0.1361	−4.6903	−0.3224

## Data Availability

No new data were created or analyzed in this study. Data sharing is not applicable to this article.
